# Ultrastructural Location and Interactions of the Immunoglobulin Receptor Binding Sequence within Fibrillar Type I Collagen

**DOI:** 10.3390/ijms21114166

**Published:** 2020-06-11

**Authors:** Jie Zhu, Rama S. Madhurapantula, Aruna Kalyanasundaram, Tanya Sabharwal, Olga Antipova, Sandra W. Bishnoi, Joseph P. R. O. Orgel

**Affiliations:** 1 Institute of Biophysics, College of science, Northwest A&F University, Yangling 712100, China; 2 Department of Biology, Illinois Institute of Technology, Chicago, IL 60616, USA; rmadhura@iit.edu (R.S.M.); sagi.aruna@gmail.com (A.K.); tanya.sabharwal@utexas.edu (T.S.); olga.antipova@gmail.com (O.A.); 3 Pritzker Institute of Biomedical Science and Engineering, Illinois Institute of Technology, Chicago, IL 60616, USA; 4 X-ray Science Division, Argonne National Laboratory, Lemont, IL 60439, USA; 5 Department of Chemistry, Illinois Institute of Technology, Chicago, IL 60616, USA; 6 Department of Biomedical Engineering, Illinois Institute of Technology, Chicago, IL 60616, USA

**Keywords:** collagen, X-ray diffraction, immunogold labeling, nanomechanical properties, MHC class I, lung disease, atomic force microscopy

## Abstract

Collagen type I is a major constituent of animal bodies. It is found in large quantities in tendon, bone, skin, cartilage, blood vessels, bronchi, and the lung interstitium. It is also produced and accumulates in large amounts in response to certain inflammations such as lung fibrosis. Our understanding of the molecular organization of fibrillar collagen and cellular interaction motifs, such as those involved with immune-associated molecules, continues to be refined. In this study, antibodies raised against type I collagen were used to label intact D-periodic type I collagen fibrils and observed with atomic force microscopy (AFM), and X-ray diffraction (XRD) and immunolabeling positions were observed with both methods. The antibodies bind close to the C-terminal telopeptide which verifies the location and accessibility of both the major histocompatibility complex (MHC) class I (MHCI) binding domain and C-terminal telopeptide on the outside of the collagen fibril. The close proximity of the C-telopeptide and the MHC1 domain of type I collagen to fibronectin, discoidin domain receptor (DDR), and collagenase cleavage domains likely facilitate the interaction of ligands and receptors related to cellular immunity and the collagen-based Extracellular Matrix.

## 1. Introduction

Collagen fibrils present in different tissues such as tendons, ligaments, bone, cornea, cartilage, skin, blood vessels, large bronchi, and lung interstitium are constituted by a hierarchical packing of collagen molecules. These fibrils are remarkably well adapted to their respective mechanical and biochemical functions. For instance, collagen fibrils and their constituent molecular components play an important role in tissue and cellular activities, including immune responses involving the major histocompatibility complex (MHC). MHC-mediated immunity involves the presentation of peptide sequences for the identification of foreign bodies or self-recognition. Under nonpathogenic circumstances, the collagen MHC peptides are recognized as self-peptides and are tolerated. In the case of autoreactivity, self-peptides, are presented to T-cells leading to autoimmune responses such as in lung fibrosis. The location of the MHC domain of type I collagen and its potential role in the organizational relationship between collagen fibrils and cellular receptors are described in this study.

### 1.1. Collagen Fibril Structure

The axial organization of collagen molecules in the fibril has a characteristic and crystalline periodic feature called the D-repeat or D-period. The D-period is composed of an overlap region (more electron-dense area with five molecules per microfibril) and gap region (less electron-dense area with four molecules per microfibril) which have differing physical characteristics due to their difference in collagen molecular composition, amino acid sequence and structure [1]. In addition to the five-to-four and overlap-to-gap ratio, the collagen molecules are essentially parallel to each other in the overlap region but adopt different molecular paths in the gap region. Furthermore, the N-, and particularly the C-terminal, telopeptide regions have increased molecular density as compared with both the gap and overlap regions, due to the relatively contracted and folded states and intermolecular cross-linking [2,3].

### 1.2. Receptor Binding with the Collagen Fibril

Various ligands bind to specific regions (i.e., domains) within the hierarchical structure of the collagen fibril. Certain ligands with immunological function seem to have clear and unobstructed access to their collagen receptor sites, such as glycoprotein VI (GPVI), Endo180 [4,5], and the human osteoclast-associated receptor (OSCAR) [6]. Others require the “removal” of specific molecular segments within the microfibrils/fibrils to reveal the underlying binding site (through peptide cleavage, damage, molecular motion, or mechanical relocation). Examples of the latter are the discoidin domain receptor (DDR), the matrix metalloproteinase (MMP), and some (but not all) integrin sites [7]. The C-terminal telopeptide region and nearby domains (described below) are the most significant matrix–matrix and matrix–cell interaction domains of the D-periodic collagen fibril [8,9]. This is partially due to this D-periodic region being the most “exposed” relative to the rest of the D-periodic structure [1,10]. It contains the C-telopeptide and cross-linking domain, the site of triple helix polymerization, and nearby, a significant fibronectin domain which overlaps a DDR binding domain and the MMP interaction domain and cleavage site [1,8].

Modeling and docking studies have indicated that a minimum of two GPO (glycine-proline-hydroxyproline) triplets are necessary for GPVI-collagen ligation [11] supported by favorable protein-protein contacts, as well as strongly supported by experimental studies [12,13]. There is just one limited region on the collagen fibril surface where two GPO repeats occur, located just prior to the non-helical C-telopeptide. This highly accessible location is also between the fibronectin/collagenase interaction domain and the start of the C-terminal telopeptide. This region is structurally exposed and prominently placed for environmental interaction, according to the collagen fibril structure first put forward by Orgel et al. [2,3,14]. It also appears to possess favorable structural characteristics for interaction with immunogloublin-like collagen receptors, such as OSCAR [6]. These receptors play in important role in the modulation of matrix remodeling and in antigen recognition [6,15]. Again, it should be noted that important domains are present in close proximity to the C-telopeptide region, such as that for collagenase cleavage, DDR, and fibronectin interaction.

The presence of these ligand-binding domains makes the C-terminal domain of the mature collagen fibril an important region for receptor mediated activation of cellular cascades, inflammation, cellular recruitment, and prevention of apoptosis.

### 1.3. Approaches to Observing Collagen Structure and Receptor Sites

Obtaining structural data for intact, hydrated collagen fibrils at sufficient resolution to distinguish between potential binding sites for receptors to the C-terminal region on the fibril surface, is challenging. Fiber X-ray diffraction remains to be the only practical method of determining the high-resolution native, in situ structure at various structural hierarchies (ultrastructural through molecular level for D-periodic fibrillar collagen). However, interpretation of data from this powerful method can involve a significant investment in experimental time and effort, whereas more accessible and commonly available techniques can produce complimentary data. Atomic force microscopy (AFM) is a particularly powerful method of detecting biological surface ultrastructure and micromechanics and possesses excellent capabilities in nanomanipulation. In this study, the relationship between structure and function, particularly between the gap and overlap regions, are considered in the context of a molecular motif probe, a monoclonal antibody against type I collagen. In the work reported here, the same antibody is used in AFM and X-ray diffraction studies to acquire confirmatory data between the techniques, while each method contributes its own significant insight. Using this combined approach, we observe, and confirm, the regular binding of the monoclonal antibody to the exterior of type I collagen fibrils maintained in their near native state. Analysis of these data confirm the crystalline condition of the collagen fibril with minimally altered mechanical behavior between control and labeled fibrils and with sufficient experimental resolution to determine which part of the D-periodic structure this anti-type I collagen antibody attaches to.

## 2. Results and Discussion

### 2.1. Elasticity and Adhesive Behavior of Collagen Fiber

**Figure 1:**
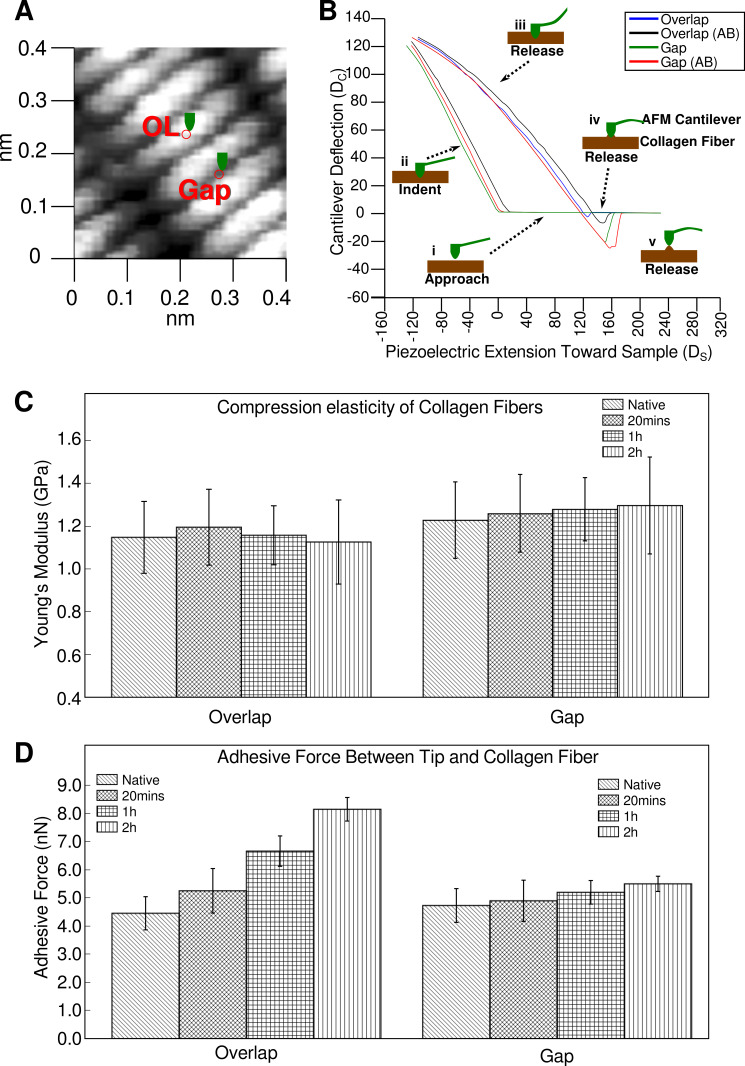
Elasticity and adhesive behavior of collagen fiber determined by atomic force microscopy (AFM) nanoindentation. (**A**) The region of interest with gap and overlap (OL) were marked where nanoindentation was performed; (**B**) Typical force-distance curves of collagen fibers recorded at overlap and gap zones in native and treated states, respectively, with a ~20 nm indentation. The plot is also annotated with the process of indentation (i–v) from the AFM tip approaching the sample through to indenting and releasing after adhesion. Data from the force curves are used to calculate transverse elasticity and adhesion force; (**C**) Transverse elasticity coefficient and Young’s modulus (*K_S_*, and *E_S_*) of native and antibody treated collagen fibers at overlap and gap zones in drying process; (**D**) Adhesion force (*F_A_*) between AFM tips and collagen fiber at overlap and gap zones with time of antibody incubation prior to data collection. Note the significant increase in adhesive force for the overlap zone of the sample treated with gold-conjugated antibody.

Figure 1 shows typical force-distance curves of collagen fibers recorded at overlap and gap zones in native and treated states, respectively, with a ∼20 nm indentation. Parameters used were μ = 0.5 by assuming the isometric changes in fibrils, and R ≈ 20 nm, K_C_ = 3.1 N/m (provided by manufacturer). The Young’s modulus of the overlap region is 1.1 ± 0.2 GPa (*n* = 40) and the gap region is 1.2 ± 0.2 GPa (*n* = 40). These measurements align with those previously determined using the nanoindentation technique (Table 1).

The differences between the Young’s moduli of the gap and overlap regions of the native collagen are small (<0.15 GPa). There were no significant differences observed with longer incubations with anti-collagen antibodies.

Significant differences are observed in the compression elasticity measurements made in the overlap regions, particularly with antibody incubation time (Table 2). The decreasing trend in error in calculation with increased incubation time indicates a gradual and uniform distribution of antibody with time in the overlap region.

There were no significant differences observed in the adhesion force in the gap region, even with longer antibody incubations (Table 3). Although a general increase in adhesion force is observed, the changes are small, especially relative to that observed in the overlap region. This indicates that the binding density of the antibody in the gap region was small and the increase in adhesion could be a result of instability and random attraction of some antibody molecules to/within the gap region.

**Figure 2:**
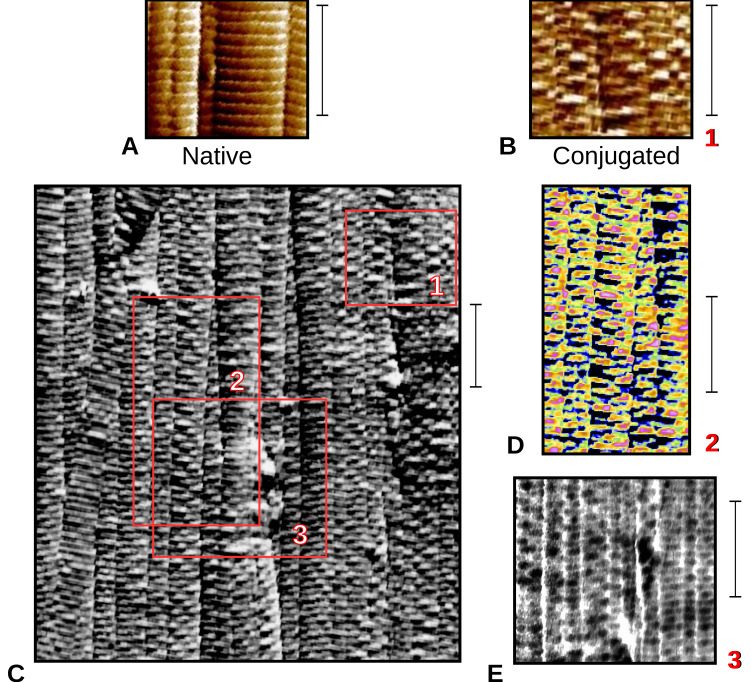
(**A**) Native collagen fibrils (amplitude/height image) for comparison with (B); (**B**) Gold-conjugated antibody (Ab) labeled collagen fibrils from region 1 of (**C**), shown at the same scale as native (unlabeled fibrils) of (A); (**C**) Area scan of Ab labeled type I collagen fibrils, AFM tapping mode, amplitude height image. 1–3 marks the positions of enlarged inserts of (**B**,**D**,**E**). (**D**) Amplitude/height (log scale). The color scale is based on height signal, and thus Ab is revealed as pink/red at the highest relative height of the scan of this area of (**C**); (**E**) Phase/height (log scale). Inverse grayscale color map, Ab appears as dark/black patches. Scale bar is ~667 nm for each part (A–E).

**Figure 3:**
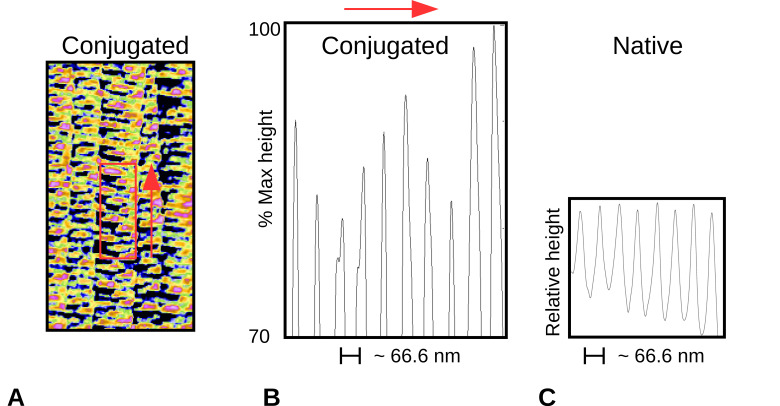
Amplitude height scan of the top 30% height along a portion of one collagen fiber. Data are consistent with one principle conjugated antibody position per D-period. (**A**) As per Figure 2D, width of red box summed to form the one-dimensional (1D) scan in the direction of the red arrow to give (B); (**B**) 1D scan of (**A**), indicates that highest regions of AFM scan (gold-conjugated antibodies that at 30 nm stand above the normal D-period collagen structure) have a principle organization consistently 1D period apart; (**C**) Native D-period scan, shows same periodicity as (**A**) and (**B**) and significantly less peak height variation.

**Figure 4:**
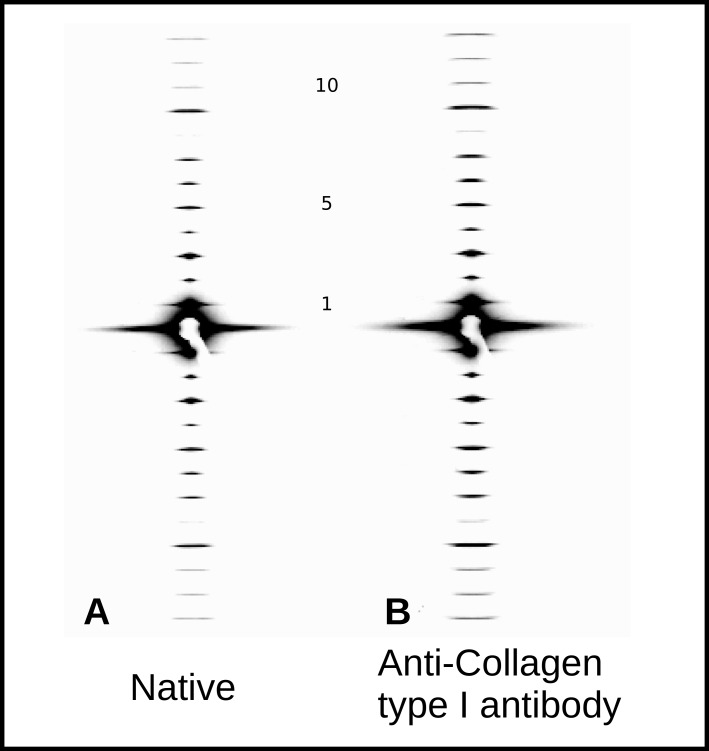
Low angle X-ray diffraction of native and antibody-labeled type I collagen tendon samples, meridional orders 1–12. Scaled to same global maximum for relative intensity comparison. (**A**) Native rat tail tendon; (**B**) Anti-collagen type I antibody-labeled rat tail tendon.

Collectively, these data indicate that the probes introduced minimal changes to the collagen fibril structure itself, other than to attach to it. This is further confirmed by the isomorphous nature of the X-ray diffraction data. That is, any significant change to the structure of the collagen fibrils that effected their ability to diffract X-rays would be apparent by either the degradation of diffraction quality or changes in the unit cell parameters leading to changes in the spacing between diffraction peaks. An isomorphous derivatization of the sample means little or no change in the position of the diffraction peaks with differences observed in diffraction intensity. In this experiment, the treatment of tendons with the antibody probe changes the intensity distribution in the diffraction pattern that originates from the unit cell (D-period) of collagen (See Figure 1, Figure 2, Figure 3 and Figure 4) [2,3,14,19,20].

Detailed statistical analyses (one-way ANOVA and multiple comparisons between and within sample groups) are reported in Supplementary Information.

### 2.2. Topography of Native Collagen Fibers

Initial experiments indicated a significant decrease in D-period length as measured by both fiber diffraction and AFM when performed out of solution or without a humid environment [3,21]. Between 1 and 10 min after removal from buffer, the D-period shrank to 66 nm (down from 67 nm). In the AFM observations, between 10 and 30 min after removal from buffer, the D-period retained a 66 nm periodicity before further shrinkage. This was an adequate interval for AFM measurements. For X-ray diffraction, the shrinkage was detectable within seconds to 10 min of removal from buffer depending on atmospheric humidity. Since it does not present a significant challenge to X-ray experiments to do so, samples remained in a buffer humid environment with a buffer bath where part of the sample was left, allowing a wick of liquid to maintain the native 67 nm structure. These differences along with resolution sensitivity, X-ray diffraction (XRD) is more sensitive than AFM in this experimental circumstance, account for the difference in working D-period lengths (67 nm XRD, 66 nm AFM, as reported here).

### 2.3. Topography of Gold-Labeled Collagen Fibers

Figure 2 shows amplitude/height AFM images on native and antibody-labeled collagen fibers. A periodic labeling by the gold-conjugated antibody is observed on closer analysis as presented (Figure 2B–E). However, banded and periodic gold particles labeling on collagen fibers has not been observed by AFM previously (the same antibody providing such consistent periodic binding that it shows in the electron density of a difference derivative in XRD, see Section 2.4). Further analyses were performed to identify the D-periodic location of the antibody labeling site on the D-period. A region of interest was identified and the peak heights in this region were summed along the width to produce a one-dimensional (1D) plot (Figure 3).

### 2.4. X-Ray Diffraction and Fourier Analyses

XRD meridional intensity data (Figure 4) were extracted from orders 1–10 and their amplitudes used to calculate 1D difference Fourier and Patterson maps. These show the antibody labeling locations and are supported by the AFM data [2,3,19,22]. The difference Fourier, using amplitudes extracted from this study and their 00L phases [2,3] indicates one major binding site with three smaller, non-zero peaks. A Patterson model calculated from the fractional positions indicated from the difference Fourier (sites at 0/0.99D, 0.31–0.33D, 0.42D–0.44D, and 0.85D) was plotted alongside the difference Patterson and found to give both non-zero peaks that the difference Patterson function does.

### 2.5. Interpretations from AFM and XRD Studies

**Figure 5:**
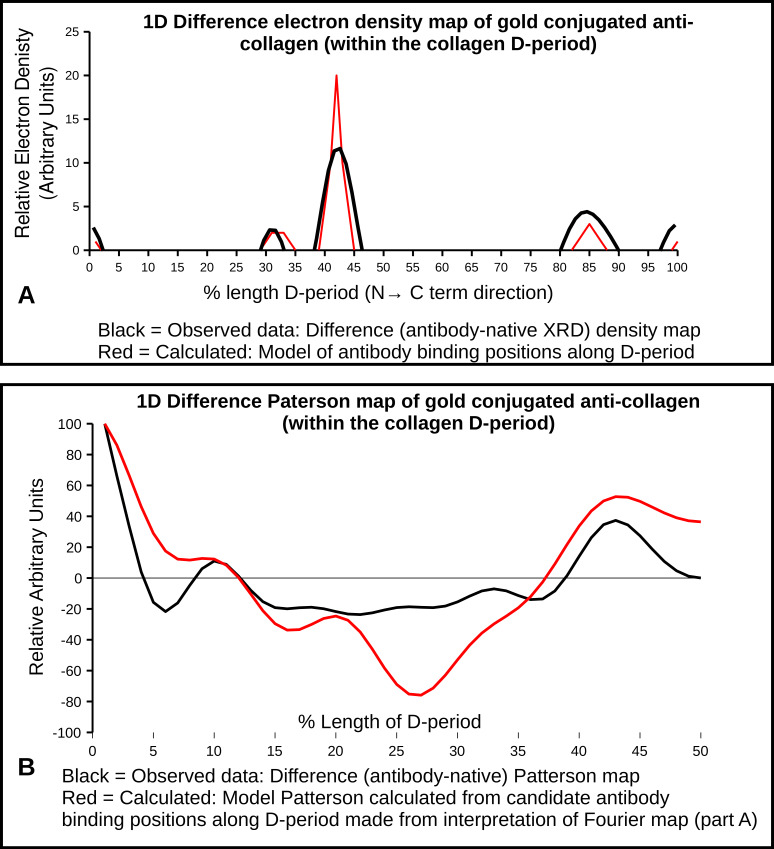
(**A**) Difference Fourier/electron density map, showing the labeling positions in the antibody treated sample; (**B**) Difference Patterson, showing the peak occurring distance functions between antibody labeling positions. There is a clear correlation between the observed and calculated Patterson, which was based entirely on the observed peaks in the difference Fourier. These were the following: 0/0.99D, 0.31–0.33D, 0.42D–0.44D, and 0.85D.

Both AFM and XRD data show D-periodic binding of the probe after 1–2 h of label incubation. AFM shows a repeating pattern (Figure 2 and Figure 3) with what appears to be a single labeling site per D-period. X-ray data appear to support this, as the difference Fourier indicates a major binding site at 0.42D and two to three minor binding sites at around 0.32, 0.85, and possibly at 0.99D (see Figure 5 and Table 4).

A derivative labeling model built on this assumption (Figure 5A), produces a Patterson that significantly resembles the difference Patterson calculated from the experimental data Figure 5B). Both show the site-to-site spacing between antibody probes within a D-period. A large non-zero peak of 0.43D and a lesser peak at 0.10D. This is the distance between 0.99/0.1 and 0.42 and 0.85D and the 0.32 and 0.42D antibody labeling sites, respectively (as indicated from the difference Fourier).

Both the Fourier and Patterson functions of the XRD data indicate that the major labeling site is at 0.42D, which is specifically at the start of the C-telopeptide. This would be the most exposed/elevated (i.e., presented on the outermost part of the fibril into the milieu of the matrix) part of the D-periodic fibril surface. This could be part of the reason why this is easily and prominently detected via AFM. This observation is in line with the site of recognition (epitope) for the antibody reported by manufacturers of anti-collagen I antibodies (COL1A1 aa. 1200–1300; see abcam Cat No. ab138492). It is interesting that this is the site of collagen polymerization and the site of GPVI and ENDO180 and OSCAR MHC1 class collagen binding molecules. Presumably this is due to the repeating modified imino peptide sequence, GPO5, at this location. This site is also neighboring (within ∼2 nm) the collagenase interaction domain and fibronectin binding domain of its nearest packing neighbor on monomer 4.

**Figure 6:**
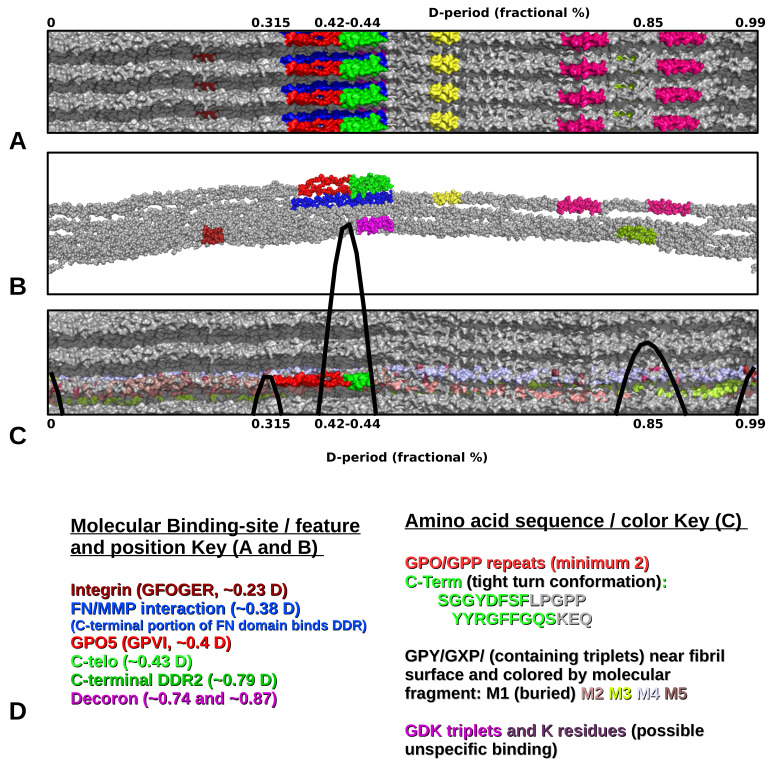
Fibril and microfibril views, colored by binding domain or amino acid features. Number scale is fractional percentage along D-period (aligned parts (A–C)). (**A**) Selected functional binding domains (fibril surface view); (**B**) Selected functional binding domains microfibril/lateral view inside fibril interior; (**C**) Amino acid sequences (fibril surface view). This includes C-telopeptide sequence in its tight turn configuration, GPO/GPP repeats, immino-rich triplets (GPY or GXP), GDK triplets, and K residues; (**D**) Color keys for parts (A–C).

As Table 4 summarizes, in the collagen fibril D-period, the antibody probe binding positions indicated by the XRD Fourier and Patterson functions are as follows:

- 0.32 which is just prior to the C-terminal telopeptide region with a cluster of modestly exposed GPP/GPR sequences on M4 and M5;
- 0.42 is at the end of the highly exposed GPO/GPP(4/5) region and the start of the non-helical telopeptide (M5), the middle of the fibronectin binding/MMP interaction domain of which the C-terminal portion is also a DDR interaction site (M4);
- 0.85 is a highly accessible area in the gap region with GPP/GAP sequences, just before the decoron D-band binding site and directly over the DDR2/von Willebrand factor (vWF) binding site, a region of known helical stability [9,22];
- 0.99–1.0/0.0 is the lowest trough of the up/down formation of the collagen fibril surface (Figure 6) and rich in lysine residues and some reoccurring GDK sequences. Its trough-like features could lead to more unspecific type interactions with the probe.

**Figure 7:**
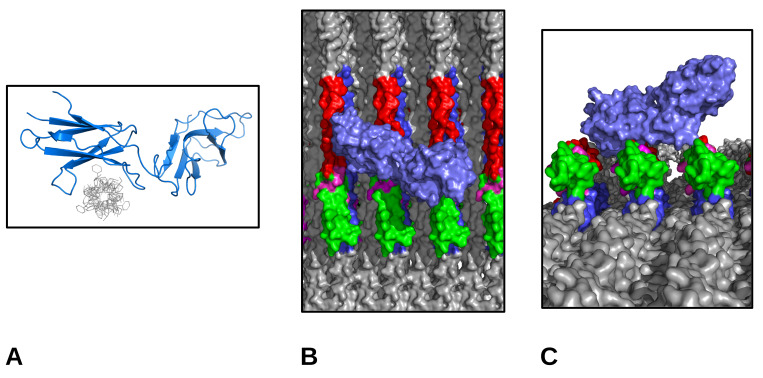
Molecular views of osteoclast-associated receptor (OSCAR) and its binding to receptor sequence of GPO repeats. (**A**) OSCAR coordinates from RCSB 5CJB (collagen-like peptide in gray, OSCAR in blue); (**B**) OSCAR receptor docked with collagen fibril surface (top view); and (**C**) As (B) but viewed along fibril surface from a point C-terminal (further into the gap region) to the visualized scene. In (A) and (B), OSCAR was docked by overlaying collagen-like peptide coordinates with the GPO sequence of one microfibril in the collagen fibril structure and rotated around the collagen molecules long axis, and then energy minimized with PyMol ”optimize” OSCAR blue-purple to clear molecular collisions. GPO repeat red, C-terminal telopeptide green. Tyrosine residues are magenta. The latter are in close proximity to GPO repeat, the exposed and elevated surface is placed for dynamic cellular interaction with obstruction from the rest of the fibrillar structure. OSCAR/GPVI receptor site on the collagen fibril is ~2 nm away from the fibronectin/the DDR overlap region site/MMP collogenolysis site.

The common theme between these sites is that GPP or GPO are closely related sequences for triple-helical stability ([23]) and repeats of GPP/GPO are the interaction sequence for these immunologically associated molecules. Regarding the major 0.42D binding site, it is interesting to note that it was previously reported that this specific antibody did not bind rat tail collagen I samples prepared via proteolytic treatment. Our data clearly show that the antibody binds (both AFM and X-ray) when the samples are left intact without removal of the telopeptides. In addition to the presence of the longest GPP/GPO repeat in the type I collagen primary structure, this could indicate a role for the C-telopeptide in interaction with the immunoglobulin or possibly, because of its tight fold confirmation [2,3], which brings several tyrosine residues into close proximity with the GPO5 repeat. Significantly, the role of tyrosine residues has been found to be highly relevant in OSCAR function. Since our methodology does not work well for non-native/non-intact preparations of fibrillar collagen, we are unable to perform an adequate control experiment to test this hypothesis directly. However, this would be almost paradoxically a negative control, that is, in using an atelo artificial fibril to replicate the non-binding result of Harris and Reiber (2007) [24] which was performed for rat skin with pepsinized collagen isolates, as opposed to intact rat tail tendon isolates used in this study. However, this speculation does not affect the observation that the binding location of the antibody is clearly shown at the interdomain location of the GPO/C-telopeptide region (Figure 6 and Figure 7).

The remaining binding sites seem comparatively minor, however, it seems noteworthy that they are in close proximity to a fibronectin binding domain (∼0.32D) and DDR binding domain (0.85D), see Figure 6 and Figure 7. However, these sites lack the exposure to the environment of the 0.42D site GPO sequence and, presumably, the triple-helical rigidity that GPO5 repeat possesses. There is evidence that the triple helices are relatively stable and well formed in these regions, with the possible exception towards the end of the α2 collagen peptide [9,22].

## 3. Materials and Methods

### 3.1. Preparation of Immunogold-Labeled Collagen Fibrils

Anti-collagen antibodies (Cat No. MAB3391; 1.5 mg/mL in 0.15 M sodium chloride, 10 mM sodium phosphate pH 7.5 with 0.1% mannitol and 0.1% Dextran) specific to rat tail tendons were obtained from Millipore Co., Temecula, CA, USA. These antibodies were used without further conjugation for XRD studies. For AFM studies, the antibodies were conjugated with 30 nm gold particles (nanoshells, Ted Pella Inc., Redding, CA, USA) using an orthopyridyl disulfide polyethylene glycol (OPSS-PEG) linker, as described by Bishnoi et al. (2011) [25]. Briefly, antibody-PEG conjugate stock was prepared by adding 10 μL of a 1.5 mg/mL solution of OPSS-PEG to 300 μL antibody solution overnight. Then, 6 μL of this stock were added to a 1 mL suspension of nanoshell and allowed to react on ice for 2.5 h. Then, 110 μL of 10-5 para-mercapto-aniline polyethylene glycol 5000 (pMA-PEG) were added to this mix and incubated for 50 min, and then centrifuged at 4000 rpm for 7 min. The supernatant was discarded, and the pellet was reconstituted with phosphate buffered saline (PBS) and stored at 4 °C until use in the experiments. This gold particle size was selected to provide the best stability with the conjugation of the antibody over a period of time sufficient to collect AFM data. A 5 nm particle was judged to be small to have confidence in either stability or later detection with AFM. A 30 nm particle provided a stable complex while still being less than half the size of the D-period to provide some manner of specificity for detecting the binding location within the D-period with AFM. The 10–20 nm particles were estimated to be not as stable for the time period required for AFM studies, but certainly could be utilized in other experiments.

Native type I collagen fibers from Wistar rat tail tendons were manually dissected using a surgical needle into the smallest possible fibers and stored in phosphate buffered saline (PBS) pH 7.4 at 4 °C for subsequent investigation. Fibers were incubated with gold-conjugated antibodies (described above) at 5 μg/mL for 20 min, 1 h, and 2 h, at 20 °C. Then, fibers were washed with PBS to remove unbound antibodies and deposited on freshly cleaved mica (Ø ≈ 1.0 cm) (Electron Microscopy Sciences Co., Hatfield, PA, USA), and stored in 12-well plates at 4 °C refrigerator for 24 h. Then, the native (untreated) and antibody-labeled preparations were washed with deionized (DI) water and dried with filter paper three times to remove PBS crystals and possible contaminants. The preparations were dried at room temperature until use for imaging and indentation studies.

### 3.2. Imaging and Topographic Analysis

Images of collagen fibers prepared above were acquired using a PicoScan-3000 AFM (Molecular Imaging Co., Tempe, AZ, USA) and Multi-Mode NanoScope(R) IIIa AFM (Veeco Metrology, Santa Barbara, CA, USA) using commercially available sharp cantilevers with a resonance frequency of 330 kHz, spring constant of 42 N/m, and tip radius of curvature R ≈ 7 nm (Nanosensors Co., Neuchatel, Switzerland). Tapping modes were used in the scanning process. Scanning force, integral and proportional gains were adjusted whenever necessary to improve the image quality, and the scanner was operated at 0.5–2 Hz in the Y direction. All experiments were carried out in the air (55% relative humidity) at room temperature. Height, deflection, and friction images were collected in 512 pixels simultaneously. AFM off-line software V5.3.3 (Molecular Imaging Co., Tempe, AZ, USA) and V5.30r3.sr3 (Veeco Metrology, Santa Barbara, CA, USA) were used to analyze the surface profiles and particle distribution in the height images by section analysis and particle analysis separately.

### 3.3. Nanoindentation and Elasticity Analysis

AFM nanoindentation of collagen fibers were carried out on a PicoScan 3000 AFM (Molecular Imaging Co., Tempe, AZ, USA). Gold-coated silicon nitride AFM cantilevers with resonance frequency of 80 kHz, spring constant of 3.1 N/m, and tip radius of curvature R ≈ 20 nm (Nanosensors Co., Neuchatel, Switzerland) were used for indentation. Collagen fibers were rastered in tapping/contact mode first, and then the regions of interest were chosen to perform nanoindentation in force mode with force-distance plots. All experiments were performed in the air (55% relative humidity) and at room temperature, and all data were shown as mean ± SD. Transverse elasticity and adhesive force at these locations were calculated from the recorded force-distance curves, as shown in Results. The indentation force (*F_S_*) applied to the sample was calculated by the cantilever deflection (*D_C_*) and its spring constant (*K_C_*). The sample deformation (*D_S_*) is equal to the piezoelectric extension (*L_P_*) from the original sample surface minus the cantilever deflection (*D_C_*) [26]. Adhesion force (*F_A_*) was read out directly from the force-distance plots, as the absolute maximum indentation force (*F_C_*). The elasticity coefficient of the sample (*K_S_*) is determined using the indentation force (*F_C_*) divided by the sample deformation (*D_S_*).

(1) $$K_{S}=\frac{F_{C}}{D_{S}}=\frac{K_{C}D_{C}}{L_{P}-D_{C}}$$

(2) $$F_{A}=\left|{\left(F_{C}\right)}_{max}\right|$$

The Young’s modulus of the sample (*E_S_*) was calculated using the Hertz formula which assumed a normal interaction of an infinitely solid spherical surface with a soft planar surface. These equations are generally better in understanding the structural and mechanical stability of soft biological samples at cellular and molecular levels. Hertz equations are as follows:

(3) $$E_{S}=\frac{3\left(1-{µ}^{2}\right)F_{C}}{4R^{1/2}D_{S}^{3/2}}=\frac{3\left(1-{µ}^{2}\right)K_{C}D_{C}}{4R^{1/2}D_{S}^{3/2}}$$

where μ is the Poisson’s ratio of the sample, which is the ratio of transverse contraction strain to longitudinal extension strain in the direction of the force and R is the radius of curvature of the AFM tip.

The data from the compression elasticity and adhesion force experiments were analyzed using SPSS 20.0 (IBM Co., Chicago, IL, USA). One-way analysis of variance (ANOVA) and multiple comparisons between and within sample groups were used to identify differences (see Supplementary Information). A difference was recorded as significant if *p* < 0.05.

### 3.4. X-Ray Diffraction

Small Angle X-ray diffraction (SAX) setup and data integration were performed at the Biophysics Collaborative Access Team (BioCAT), at Argonne National Laboratory, Chicago, IL, USA, as reported previously [19]. Approximately 15 orders of diffraction of the fibrillar collagen type I meridional pattern were recorded for the control (native) and the antibody-labeled rat tail tendons. The amplitudes (square root of intensity) of orders 1–10 (to give an isomorphous resolution of ∼6.7 nm) were scaled after those reported in Antipova and Orgel 2010 [18]. The native rat tail tendon data were subtracted from the antibody-labeled rat tail tendons data and this difference derivative data combined with the native collagen phases were used to calculate a difference Fourier map of 00L = 1–10 (after Orgel et al. (2000), Antipova and Orgel (2010), Orgel et al. (2014) [3,19,20]). We note that isomorphous labeling can only likely occur upon the surface of the fibril/s which reduces the maximum possible signal from difference Fourier or Patterson’s relative to full volume of the fibril.

### 3.5. Molecular Visualization

Molecular visualization was performed as previously described [7,9,10,20,27]. Molecular surfaces were rendered with PyMol and key receptor binding sequences/features color coded for ease of reference. The collagen molecular coordinates were from 3HR2 and OSCAR from 5CJB of the RCSB database [28].

OSCAR was docked by overlaying molecular coordinates from a collagen-like peptide from 5CJB with the fibril surface structure composed from 3HR2, and the GPO sequence of one microfibril in the collagen fibril structure. OSCAR was rotated around the collagen molecules long axis while maintaining contact with the binding surfaces, and then energy minimized with PyMol’s ”optimize” (local optimization, 1000 steps, MMFF94s, conjugate gradients reaching −1524 Kcal/mol) to clear molecular collisions.

## 4. Immunoglobulin-Like Collagen Receptor—Conclusions

Previously, it has been observed that the GPO5 repeat sequence is, unlike other collagen-receptor sequences in the fibril, very highly accessible (Figure 7) [20,29]. As with GPVI receptor binding, the collagen binding sequence of OSCAR allows the receptor to dock with the fibril without any obstructions. It is interesting to note, the importance of the tyrosine residues of the receptor to binding the GPO sequence [15]. It is intriguing that a concentration of a few tyrosine residues in the C-terminal telopeptide is the only place tyrosine residues are found in such concentration (if at all) in the type I collagen sequence. In fact, the C-telopeptide is held in a tight turn configuration to bring all of these tyrosine residues into one closely spaced region [2,3]. However, the physiological relevance of this concentration, in close proximity to the GPO repeat sequence, remains to be explored. The three-dimensional (3D) folding structure assumed by the C-telopeptide to bring all of these tyrosine residues into close proximity to the GPO5 sequence is interesting, but it is not clear as to what role it could play, beyond of course, the possibility of structural stability to what could be a highly important region of the collagen fibril to cellular attachment and activation.

An example of such is OSCAR, an immunoglobulin-like activating receptor of the leukocyte complex [6,15]. It is expressed at high levels in bone resorbing cells, where its binding to collagen results in several activated signaling pathways involving matrix remodeling and immune cell function. OSCAR has also been found to be expressed in vascular endothelial cells, macrophages, neutrophils, and monocytes. Thus, it is likely to fulfill a variety of roles beyond just adhesion and activation [30]. Due to it being a immunoglobulin-like collagen-binding receptor and because of its recent structural characterization complexed with a collagen-like protein, OSCAR was chosen as a docking candidate to illustrate the free accessibility of the GPO5/C-telo domain region. Our data indicate that this same domain is the major binding site of the monoclonal collagen antibody. Thus, due to a common ligand binding sequence and the observed co-location of antibody binding and specific GPP/GPO repeats in the collagen fibril, it would seem likely that binding between these immunoglobulin-like collagen receptors and the collagen monoclonal antibody are not unrelated to that shown here.

## Figures and Tables

**Table 1:** Young’s Modulus of the collagen determined by AFM nanoindentation, in comparison to the results reported here.

	Young’s Modulus (GPa)	Source Tissue
Results reported here	1.1 ± 0.2 (overlap) 1.2 ± 0.2 (gap)	Rat tail tendon
Heim et al., 2006 [16]	1–2	Inner dermis of sea cucumber
Strasser et al., 2007 [17]	~1.2	Calf skin
Grant et al., 2007 [18]	1.9 ± 0.5	Bovine achilles tendon

**Table 2:** Adhesion force measurements form the overlap region after antibody treatment.

Incubation Time	*n*	Adhesion Force (nN)	Percentage Error (%)	Percentage Change from Native (%)
Native (0 min)	40	4.5 ± 0.6	13.3	-
20 min	40	5.3 ± 0.8	15.1	17.8
1 h	40	6.7 ± 0.5	7.5	48.9
2 h	40	8.2 ± 0.4	4.9	82.2

**Table 3:** Adhesion force measurements form the gap region after antibody treatment.

Incubation Time	*n*	Adhesion Force (nN)	Percentage Error (%)	Percentage Change from Native (%)
Native (0 min)	40	4.8 ± 0.6	12.5	-
20 min	40	5.0 ± 0.7	14.0	4.2
1 h	40	5.3 ± 0.4	7.5	10.4
2 h	40	5.6 ± 0.2	3.6	16.6

**Table 4:** Antibody binding positions.

Fractional Peak Position	Fibril Accessibility	Candidate Amino Acid Sequences (Monomer (M) and Sequence)	Functional Binding Region of Collagen D-Period
0.31–0.33	Just prior to C-term telopeptide. M4, M5	M4 GPP(2) M5 GPPGPR	Near (N-terminal) fibronectin binding site (M4)
0.42–0.44	Interdomain, helical/non-helical of M5 near C-term telopeptide. M3, M4, M5	M4 GVVGLPGQRGER M5 GPO(orP)(5) SGY/YYR	Middle of the fibronectin binding/MMP interaction domain (M4)
0.84–085	Highly accessible area in gap region, just before (*n*-terminal) decoron d-band binding site	M4 GPP(GAP)3GPVGPA	Directly over DDR/VWF on M3 and adjacent to decoron binding on M4
0.99–0.1	Lowest trough of the up/down formation of the collagen fibril surface, near N-term telopeptide	M4 GPRGDK/GSRGAAGPP M5 GPRGDK/GETGEQ	Near N-terminal telopeptide, possible decorin binding region

## Supplementary Materials

The following are available online at https://www.mdpi.com/1422-0067/21/11/4166/s1.

## Abbreviations

AFM	Atomic force microscopy
XRD	X-ray diffraction
MHC	Major histocompatibility complex
DDR	Discoidin domain receptor
ECM	Extracellular Matrix
GPP	Glycine – Proline – Proline
GPR	Glycine – Proline – Arginine
GPVI	Glycoprotein VI
MMP	Matrix metalloproteinase
OSCAR	Human osteoclast-associated receptor
GPO	Glycine-proline-hydroxyproline
OPSS PEG	Orthopyridyl disulfide polyethylene glycol
pMA-PEG	Para-mercapto-aniline polyethylene glycol 5000
PBS	Phosphate buffered saline
DI Water	Deionized water
vWF	Von Willebrand Factor
