# Supplementary material for: Ultrastructural Location and Interactions of the Immunoglobulin Receptor Binding Sequence within Fibrillar Type I Collagen
Source: Int J Mol Sci. 2020 Jun 11;21(11):4166. doi: 10.3390/ijms21114166 (PMC7312686; doi:10.3390/ijms21114166)
Supplement: Supplementary file 1 [file ijms-21-04166-s001.pdf]

International Journal of Molecular Sciences

*Article*

# Supplementary Information

## S1. P-values from one-way ANOVA and multiple comparisons on and between sample groups

p-values from statistical analyses performed using one-way ANOVA and multiple comparisons between sample groups are presented in Table S1. More information on these statistics can are presented in Supplemental Information.

Table S1: p-values from statistical analyses performed on Young's Modulus and Adhesion Force, by sample and between sample groups.

| | One-way ANOVA | Multiple comparisons to native | | |
|---|---|---|---|---|
| | | 20min | 1h | 2h |
| Young's Modulus | | | | |
| Overlap | 0.187 | 0.136 | 0.825 | 0.517 |
| Gap | 0.235 | 0.397 | 0.159 | 0.049 |
| Adhesive Force | | | | |
| Overlap | 0.000 | 0.001 | 0.001 | 0.001 |
| Gap | 0.000 | 0.001 | 0.001 | 0.001 |

## S2. Statistical analyses on the Young's modulus of the overlap region of the native and antibody-treated collagen fibrils

### *S2.1 One-way ANOVA*

Descriptives
Young's Modulus of Overlap

| | N | Mean | Std. Deviation | Std. Error | 95% Confidence Interval for Mean | |
|---|---|---|---|---|---|---|
| | | | | | Lower Bound | Upper Bound |
| 0 | 40 | 1.1428 | .14000 | .02214 | 1.0980 | 1.1875 |
| 20 | 40 | 1.1918 | .15040 | .02378 | 1.1436 | 1.2399 |
| 60 | 40 | 1.1500 | .12009 | .01899 | 1.1116 | 1.1884 |
| 120 | 40 | 1.1215 | .17027 | .02692 | 1.0670 | 1.1760 |
| Total | 160 | 1.1515 | .14717 | .01163 | 1.1285 | 1.1745 |

ANOVA
Young's Modulus of Overlap

| | Sum of Squares | df | Mean Square | F | Sig. |
|---|---|---|---|---|---|
| Between Groups | .104 | 3 | .035 | 1.619 | .187 |
| Within Groups | 3.340 | 156 | .021 | | |
| Total | 3.444 | 159 | | | |

*S2.2 Post Hoc Tests*

Multiple Comparisons
Young's Modulus of Overlap
LSD

| (I) binding time | (J) binding time | Mean Difference (I-J) | Std. Error | Sig. | 95% Confidence Interval | |
|---|---|---|---|---|---|---|
| | | | | | Lower Bound | Upper Bound |
| 0 | 20 | -.04900 | .03272 | .136 | -.1136 | .0156 |
| | 60 | -.00725 | .03272 | .825 | -.0719 | .0574 |
| | 120 | .02125 | .03272 | .517 | -.0434 | .0859 |
| 20 | 0 | .04900 | .03272 | .136 | -.0156 | .1136 |
| | 60 | .04175 | .03272 | .204 | -.0229 | .1064 |
| | 120 | .07025* | .03272 | .033 | .0056 | .1349 |
| 60 | 0 | .00725 | .03272 | .825 | -.0574 | .0719 |
| | 20 | -.04175 | .03272 | .204 | -.1064 | .0229 |
| | 120 | .02850 | .03272 | .385 | -.0361 | .0931 |
| 120 | 0 | -.02125 | .03272 | .517 | -.0859 | .0434 |
| | 20 | -.07025* | .03272 | .033 | -.1349 | -.0056 |
| | 60 | -.02850 | .03272 | .385 | -.0931 | .0361 |

*. The mean difference is significant at the 0.05 level.

**S3. Statistical analyses on the Young's modulus of the gap region of the native and antibody-treated collagen fibrils**

*S3.1 One-way ANOVA*

Descriptives
Young's Modulus of Gap

| | N | Mean | Std. Deviation | Std. Error | 95% Confidence Interval for Mean | |
|---|---|---|---|---|---|---|
| | | | | | Lower Bound | Upper Bound |
| 0 | 40 | 1.2200 | .15004 | .02372 | 1.1720 | 1.2680 |
| 20 | 40 | 1.2503 | .16074 | .02542 | 1.1988 | 1.3017 |
| 60 | 40 | 1.2705 | .13066 | .02066 | 1.2287 | 1.3123 |
| 120 | 40 | 1.2908 | .19030 | .03009 | 1.2299 | 1.3516 |
| Total | 160 | 1.2579 | .16005 | .01265 | 1.2329 | 1.2829 |

ANOVA
Young's Modulus of Gap

| | Sum of Squares | df | Mean Square | F | Sig. |
|---|---|---|---|---|---|
| Between Groups | .109 | 3 | .036 | 1.434 | .235 |
| Within Groups | 3.964 | 156 | .025 | | |
| Total | 4.073 | 159 | | | |

*S3.2 Post Hoc Tests*

Multiple Comparisons
Young's Modulus of Gap
LSD

| (I) binding time | (J) binding time | Mean Difference (I-J) | Std. Error | Sig. | 95% Confidence Interval | |
|---|---|---|---|---|---|---|
| | | | | | Lower Bound | Upper Bound |
| 0 | 20 | -.03025 | .03564 | .397 | -.1007 | .0402 |
| | 60 | -.05050 | .03564 | .159 | -.1209 | .0199 |
| | 120 | -.07075* | .03564 | .049 | -.1412 | -.0003 |
| 20 | 0 | .03025 | .03564 | .397 | -.0402 | .1007 |
| | 60 | -.02025 | .03564 | .571 | -.0907 | .0502 |
| | 120 | -.04050 | .03564 | .258 | -.1109 | .0299 |
| 60 | 0 | .05050 | .03564 | .159 | -.0199 | .1209 |
| | 20 | .02025 | .03564 | .571 | -.0502 | .0907 |
| | 120 | -.02025 | .03564 | .571 | -.0907 | .0502 |
| 120 | 0 | .07075* | .03564 | .049 | .0003 | .1412 |
| | 20 | .04050 | .03564 | .258 | -.0299 | .1109 |
| | 60 | .02025 | .03564 | .571 | -.0502 | .0907 |

*. The mean difference is significant at the 0.05 level.

**S4. Statistical analyses on the Adhesion force of the overlap region of the native and antibody-treated collagen fibrils**

*S4.1 One-way ANOVA*

Descriptives
Adhesive Force of Overlap

| | N | Mean | Std. Deviation | Std. Error | 95% Confidence Interval for Mean | |
|---|---|---|---|---|---|---|
| | | | | | Lower Bound | Upper Bound |
| 0 | 40 | 4.5000 | .60000 | .09487 | 4.3081 | 4.6919 |
| 20 | 40 | 5.3000 | .80224 | .12685 | 5.0434 | 5.5566 |
| 60 | 40 | 6.7000 | .50230 | .07942 | 6.5394 | 6.8606 |
| 120 | 40 | 8.2000 | .40064 | .06335 | 8.0719 | 8.3281 |
| Total | 160 | 6.1750 | 1.53192 | .12111 | 5.9358 | 6.4142 |

ANOVA
Adhesive Force of Overlap

| | Sum of Squares | df | Mean Square | F | Sig. |
|---|---|---|---|---|---|
| Between Groups | 317.900 | 3 | 105.967 | 299.254 | .000 |
| Within Groups | 55.240 | 156 | .354 | | |
| Total | 373.140 | 159 | | | |

*S4.2 Post Hoc Tests*

Multiple Comparisons
Adhesive Force of Overlap
LSD

| (I) binding time | (J) binding time | Mean Difference (I-J) | Std. Error | Sig. | 95% Confidence Interval | |
|---|---|---|---|---|---|---|
| | | | | | Lower Bound | Upper Bound |
| 0 | 20 | -.80000* | .13306 | .000 | -1.0628 | -.5372 |
| | 60 | -2.20000* | .13306 | .000 | -2.4628 | -1.9372 |
| | 120 | -3.70000* | .13306 | .000 | -3.9628 | -3.4372 |
| 20 | 0 | .80000* | .13306 | .000 | .5372 | 1.0628 |
| | 60 | -1.40000* | .13306 | .000 | -1.6628 | -1.1372 |
| | 120 | -2.90000* | .13306 | .000 | -3.1628 | -2.6372 |
| 60 | 0 | 2.20000* | .13306 | .000 | 1.9372 | 2.4628 |
| | 20 | 1.40000* | .13306 | .000 | 1.1372 | 1.6628 |
| | 120 | -1.50000* | .13306 | .000 | -1.7628 | -1.2372 |
| 120 | 0 | 3.70000* | .13306 | .000 | 3.4372 | 3.9628 |
| | 20 | 2.90000* | .13306 | .000 | 2.6372 | 3.1628 |
| | 60 | 1.50000* | .13306 | .000 | 1.2372 | 1.7628 |

*. The mean difference is significant at the 0.05 level.

**S5. Statistical analyses on the Adhesion force of the gap region of the native and antibody-treated collagen fibrils**

*S5.1 One-way ANOVA*

Descriptives
Adhesive Force of Gap

| | N | Mean | Std. Deviation | Std. Error | 95% Confidence Interval for Mean | |
|---|---|---|---|---|---|---|
| | | | | | Lower Bound | Upper Bound |
| 0 | 40 | 4.8000 | .60383 | .09547 | 4.6069 | 4.9931 |
| 20 | 40 | 5.0000 | .70274 | .11111 | 4.7753 | 5.2247 |
| 60 | 40 | 5.3000 | .40000 | .06325 | 5.1721 | 5.4279 |
| 120 | 40 | 5.6000 | .20631 | .03262 | 5.5340 | 5.6660 |
| Total | 160 | 5.1750 | .59389 | .04695 | 5.0823 | 5.2677 |

ANOVA
Adhesive Force of Gap

| | Sum of Squares | df | Mean Square | F | Sig. |
|---|---|---|---|---|---|
| Between Groups | 14.700 | 3 | 4.900 | 18.473 | .000 |
| Within Groups | 41.380 | 156 | .265 | | |
| Total | 56.080 | 159 | | | |

*S5.2 Post Hoc Tests*

Multiple Comparisons
Adhesive Force of Gap
LSD

| (I) binding time | (J) binding time | Mean Difference (I-J) | Std. Error | Sig. | 95% Confidence Interval | |
|---|---|---|---|---|---|---|
| | | | | | Lower Bound | Upper Bound |
| 0 | 20 | -.20000 | .11516 | .084 | -.4275 | .0275 |
| | 60 | -.50000* | .11516 | .000 | -.7275 | -.2725 |
| | 120 | -.80000* | .11516 | .000 | -1.0275 | -.5725 |
| 20 | 0 | .20000 | .11516 | .084 | -.0275 | .4275 |
| | 60 | -.30000* | .11516 | .010 | -.5275 | -.0725 |
| | 120 | -.60000* | .11516 | .000 | -.8275 | -.3725 |
| 60 | 0 | .50000* | .11516 | .000 | .2725 | .7275 |
| | 20 | .30000* | .11516 | .010 | .0725 | .5275 |
| | 120 | -.30000* | .11516 | .010 | -.5275 | -.0725 |
| 120 | 0 | .80000* | .11516 | .000 | .5725 | 1.0275 |
| | 20 | .60000* | .11516 | .000 | .3725 | .8275 |
| | 60 | .30000* | .11516 | .010 | .0725 | .5275 |

*. The mean difference is significant at the 0.05 level.
